# Semantic Data Visualisation for Biomedical Database Catalogues †

**DOI:** 10.3390/healthcare10112287

**Published:** 2022-11-15

**Authors:** Arnaldo Pereira, João Rafael Almeida, Rui Pedro Lopes, José Luís Oliveira

**Affiliations:** 1 Department of Electronics, Telecommunications and Informatics/Institute of Electronics and Informatics Engineering of Aveiro, Intelligent Systems Associate Laboratory, University of Aveiro, 3810-193 Aveiro, Portugal; 2 Department of Computation, University of A Coruña, 15071 A Coruña, Spain; 3 Research Centre in Digitalization and Intelligent Robotics, Polytechnic Institute of Bragança, 5300-253 Bragança, Portugal

**Keywords:** evidence-based medicine, biomedical data, semantic data, temporal data, visualisation techniques, data visualisation

## Abstract

Biomedical databases often have restricted access policies and governance rules. Thus, an adequate description of their content is essential for researchers who wish to use them for medical research. A strategy for publishing information without disclosing patient-level data is through database fingerprinting and aggregate characterisations. However, this information is still presented in a format that makes it challenging to search, analyse, and decide on the best databases for a domain of study. Several strategies allow one to visualise and compare the characteristics of multiple biomedical databases. Our study focused on a European platform for sharing and disseminating biomedical data. We use semantic data visualisation techniques to assist in comparing descriptive metadata from several databases. The great advantage lies in streamlining the database selection process, ensuring that sensitive details are not shared. To address this goal, we have considered two levels of data visualisation, one characterising a single database and the other involving multiple databases in network-level visualisations. This study revealed the impact of the proposed visualisations and some open challenges in representing semantically annotated biomedical datasets. Identifying future directions in this scope was one of the outcomes of this work.

## 1. Introduction

The secondary use of health data is a common strategy in medical research to conduct observational studies in various domains [1], ranging from pharmacological research to public health policy design [2]. Several steps are necessary to plan and execute this type of study successfully. The first step is to define the research question, focusing on solving the problems. Therefore, the cohort group is established with the inclusion and exclusion criteria and the outputs to be evaluated. After formalising the study protocol, researchers must identify relevant information resources and possible data partners. This is accomplished by contacting the database owners to guarantee a data access agreement, analysing the data and publishing the research results [3]. Across this process, identifying databases of interest is a critical step in conducting high-quality observational studies. Therefore, solutions that simplify the searching process for biomedical databases may help researchers at this stage.

Biomedical data are prevalent in the international scientific landscape, enabling the creation of numerous repositories and databases published online [4]. For the benefit of researchers who need these sources of information to conduct their research, mechanisms to find data are required. Although retrieving information from web pages using search engines has been a common practice for many years, the same solution for data repositories is still in an early stage [5]. Another way is to use database catalogues, where data owners can publish characteristics about their databases [6]. A catalogue usually contains metadata describing databases, leading to communities of researchers sharing knowledge in a common domain of interest. We can identify some illustrative examples in biomedicine. Cafe Variome https://www.cafevariome.org/ (accessed on 14 September 2022), for instance, enables data discovery based on the semantic similarity of diseases, phenotypes and drugs, relating patient data to the terms of an ontology [7]. YummyData http://yummydata.org/ (accessed on 14 September 2022) monitors SPARQL endpoints to collect biomedical linked data [8]. FAIRsharing https://fairsharing.org/ (accessed on 14 September 2022) is another resource that describes and links data policies, repositories and databases, with a strong focus on the natural sciences [9].

Database catalogues make information accessible from a centralised access point. However, sometimes it is still not trivial to choose the best data sources. When one has multiple databases that meet different requirements, performing more specific studies may be complex, such as conducting patient-level prediction studies [10]. In these studies, researchers need to identify the datasets used to train the prediction models and the datasets used to validate the predictions [11]. These decisions usually involve a deeper understanding of the datasets publicised by the community. It is desirable to lighten the burden of these choices by having some recommendation mechanism or user-friendly interfaces allowing the data to be queried in a way that is both simple to use and that provides non-trivial results [12].

When using a recommendation system, recommendations are based on rules that can be more or less adaptive to new situations [13]. While history-based learning, when using these tools, can lead to better results over time, it is still not the best approach for many cases. We can build queries using forms that combine the descriptive metadata of the datasets of interest for better results. However, creating more complex queries is not always easy. Natural language interfaces can make searches much easier [14], but reading the results may not be intuitive enough, even with a good search strategy.

The presentation of semantic search results for metadata is usually reduced to simple tabular data [7,8,9]. However, presenting tabular data does not take advantage of the relationships that link the various entities. Graphs are another common way of presenting semantic data [15], but they may not provide the best information for extensive graphs. On the other hand, we also want mechanisms that allow browsing the data and possibly building new queries.

Our work aims to improve decision making by medical researchers using biomedical database catalogues to support the realisation of medical studies. Considering that the usual method of visualisation in table format is prevalent in this kind of solution, we propose a wide range of semantic metadata visualisations for several common challenges when selecting databases to conduct medical studies. The representation of this metadata can optimise the process of selecting data sources and refining the study scope during the study’s feasibility stage. The selected views were discussed between data partners and researchers in previous European projects.

This paper is an extension of work presented at the 35th IEEE International Symposium on Computer-Based Medical Systems (IEEE CBMS2022) [16]. We added five more semantic data visualisations in the context of exploring biomedical database catalogues. This analysis identifies points to improve in a catalogue that publishes metadata from multiple health databases, exemplifying the transverse limitations of the most common catalogues. Following this analysis, we show possible semantic information visualisations in different health contexts. This work aimed to understand the best strategies to represent data applied to this domain and identify the open challenges for the better representation of biomedical datasets.

## 2. Background

This section addresses the visualisation and comparison of semantic data and the problem of criteria changing for the inclusion or exclusion of elements in an observational study.

### 2.1. Querying and Visualisation of Semantic Data

The visualisation of semantic data ranges from the simple organisation of semantic triples in tables to the visualisation of graphs, taking advantage of the relationships between the different entities present. This last form allows richer visualisations; still, it is not uncommon to fall into scenarios where a high number of entities and relationships prevents a clear reading by users. Some examples of solutions for querying and visualising semantic data can be presented. Yet Another SPARQL GUI (YASGUI) https://github.com/TriplyDB/Yasgui (accessed on 14 September 2022) is a SPARQL client that uses module tabs to allow independent access to multiple endpoints [17]. The tool is packaged with autocomplete support, syntax checking, syntax highlighting, query sharing, query retention, and file upload/download.

SPARQLGraph https://github.com/tadKeys/sparqlgraph (accessed on 14 September 2022) is a web-based platform implemented using the diagramming library mxGraph https://jgraph.github.io/mxgraph/ (accessed on 14 September 2022) for graphically querying biological semantic databases [18]. Users can compose graph queries on a drawing board by adding new visual elements (nodes and edges). Users can only choose between elements resulting from a previous choice made by the tool’s creators, which is a limitation.

The PIBAS FedSPARQL https://github.com/marijadjokic/PIBASFedSPARQL (accessed on 14 September 2022) [19] solution was applied to a use case where data are collected from tests with bioactive substances and annotated against an ontology. The proposed solution enables the federation of those data with external information that can be extracted from global initiatives such as Bio2RDF [20], Chem2Bio2RDF [21], and the EMBL-EBI platform [22]. The system offers templates and generates static federated SPARQL queries for the retrieval of relevant information. The results are presented in tabular form.

Lekschas et al. [23] proposed SATORI https://satori.lekschas.de/ (accessed on 14 September 2022), an integrative search and visual exploration interface for biomedical data repositories. It allows performing ontology-guided visual exploration, enabling researchers to search, browse and semantically query data repositories seamlessly. The solution is based on a fixed list of datasets and does not automatically incorporate a methodology to infer structural information (ontology). Neither can it connect to an arbitrary SPARQL endpoint, immediately starting to navigate the data.

Semantic data have a graph or network structure that allows graphic visualisations, emphasising the relationships between the various entities present. We can directly use elaborate and high-level programming languages with abstraction layers to construct graphs, establishing a balance between expressiveness and ease of programming. The lower the degree of abstraction, the greater the requirement for programming knowledge and the lower the productivity. We can capture some data subtleties with lower-level programming, which otherwise might go unnoticed. However, visualisation libraries are naturally used in most applications because they provide convenient resources for various applications [24].

Despite the various display options presented, limitations remain. There are strategies to create different dashboards to visualise data and compare them. However, regarding semantic data, the availability of filters is more limited, for example, when a researcher wants to select a set of databases to answer the research questions and notices that the initial cohort is not the most suitable for the study to be carried out, or when adjusting some of the returned parameters according to specific needs, such as adding a certain threshold. Another typical situation is when choosing a set of patients suffering from a particular disease or taking a specific drug. It is necessary to navigate the underlying ontology by visually adjusting the parameters to generate new data queries.

### 2.2. Interacting with Semantic Data Visualisations

A visual query system (VQS) is a non-formal solution to database querying that uses visual representations to depict the domain of interest and help express knowledge base queries. VQS can be divided into form-based, diagram-based, icon-based, faceted, and hybrid systems A form (e.g., a table) is a named collection of objects with the same structure and is the most basic approach after plain text use. Diagram-based representations (e.g., a graph) allow exploring and showing relationships between entities. Icons denoting entities allow performing queries by combinations according to some spatial syntax on icon-based solutions. Faceted systems use classifications that organise items into multiple independent views. Solutions that combine more than one visual representation are called hybrid solutions [25,26].

Proper development of information systems depends on understanding users’ needs. It is necessary to have exploratory search mechanisms to use data repositories helpfully. As highlighted by Marchionini et al. [27], short queries typed into search boxes do not fulfil current users’ needs. Compared to analytical search strategies that depend on a carefully planned series of questions, browsing depends on on-the-fly choices, encompassing selection, navigation, and most importantly, trial-and-error tactics. We also expect to see query suggestions. This dynamic behaviour poses many challenges because the system should follow users’ expectations, providing rich interactive features.

Strictly related to user interfaces allowing working at multiple levels of detail, Cockburn et al. [28] identified four approaches: overview + detail, zooming, focus + context, and cue-based techniques. The overview + detail approach creates a spatial separation between contextual and detailed information. Zooming allows perspectives with varying degrees of proximity to the objects of interest (with temporal separation between the views). Focus + context allows integrating both focus and context into a single display. Finally, cue-based depicts the elements modified to highlight, suppress, or contextualise them.

In general, information visualisation has two main dimensions: representation and interaction. There are many possibilities for graph representation, such as arc diagrams, area grouping, centralised bursts, radial convergence, centralised rings, circled globes, circular ties, spheres, and scaling circles [29]. In arc diagrams, we use a single axis to display all nodes and semicircles to represent the arcs. Area grouping makes clusters of interconnected nodes evident, and a centralised burst highlights important nodes identifiable as highly connected. Radial convergence allows visualising relations between nodes arranged in a circle. We can use a centralised ring to check the relationship of multiple nodes with a single central node. Circled globes are projections of other topologies on a globe. Circular ties connect several centralised rings to a central node. It can be helpful to have nodes and arcs drawn on spheres. Scaling circles allow the aggregation of similar nodes.

Yi et al. [30] and Heer et al. [31] pointed out the following guidelines for interaction techniques: visualise data by choosing visual encodings, organise multiple windows and workspaces, select and mark something as interesting, explore (navigate) to show something else, reconfigure to deliver a different arrangement, encode to offer a diverse depiction, abstract to see more or less detail, filter to see something conforming to a condition, coordinate to see related items, sort items to expose patterns, derive values or models from source data, record analytics history, and annotate patterns to document findings.

An interaction must adapt to the particular problem to be solved, and it organises around a user’s intent, hiding the system’s low-level interaction details. In our context, visualisation means choosing the application layout that best fits users’ intents. It ties in closely with organising multiple windows and workspaces. The “select” feature allows users to mark and track items of interest, such as nodes and edges. When exploring (navigating), users want to examine a different subset of data cases to gain understanding and insight. The abstraction/elaboration interaction provides the necessary level adjustment of a data representation, while the filtering can reduce the representation’s complexity by hiding the elements that are not relevant to the user. The “connect” primitive traces the same object when presented simultaneously in different views. We use the sorting operation to surface trends or organise data around some analysis unit. Through their actions, users create imminently unrepeatable hypotheses and generate chains of queries the app must save for future use. The “record” and “annotate” features are helpful to deal with that issue. “Encoding” allows changes in the visual appearance of each data element, such as changing size or shape. In a more general way, the reconfiguration feature must provide users with different perspectives to uncover hidden characteristics of nodes and their relations.

Users’ behaviour is iterative and depends on their cognitive style [32]. However, when referring to usability, we are not interested in aspects belonging to the domain of behavioural sciences but in the scientific understanding of usability based on experimental data. Evaluation is essential to assess the system’s relative success compared with others. Elbedweihy et al. [33] overviewed semantic search evaluation initiatives, pointing out the importance of considering information retrieval evaluation activities in general. We want to know how users’ search requests are handled by performing a system-oriented evaluation. Equally crucial are user-oriented assessments. We can highlight efficiency, learnability, utility, and user satisfaction. Typical assessment tools include event logs, think-alouds, and questionnaires. As a final note, we register that Hilbert et al. [34] extensively studied the extraction of usability information from user interface events by processing logs.

We can intuitively build queries when using a query builder with visual artefacts. However, it is necessary to go further and obtain visualisations that allow us to reissue questions and manipulate the results in a flexible way that will enable comparisons and refinements (e.g., to redefine cohorts).

## 3. Materials

The European Medical Information Framework (EMIF) http://www.emif.eu (accessed on 14 September 2022) initiative focused on creating a European Medical Information Framework to provide better healthcare using the vast amounts of biomedical data available. A web platform was thus designed to offer a database catalogue (the EMIF Catalogue https://emif-catalogue.eu/) (accessed on 14 September 2022) where data custodians can publish metadata about their biomedical databases with different levels of granularity. The EMIF Catalogue also enables the creation of communities that gather around common interests to share and have access to convenient biomedical data [35].

**Figure 1:**
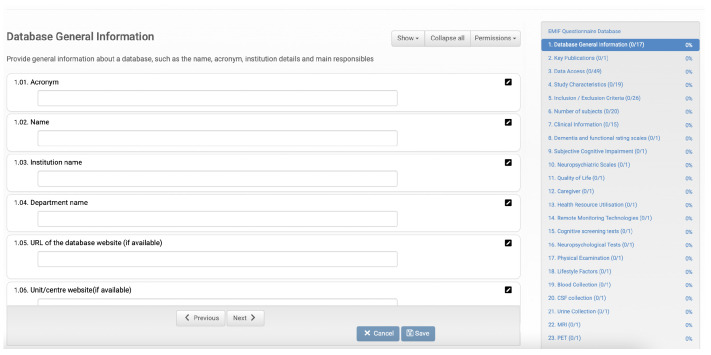
EMIF database questionnaire form to collect database characteristics. On the left, we have several fields to be filled in, and on the right, the different categories of data to be entered together with the current filling status.

For each database described in the catalogue, the data custodian must provide information that constitutes the database fingerprint (Figure 1). This information has several fields, such as the name of the database, identification of the institution that owns it, its location, and the person in charge, among many others, defined collaboratively by the community members. The fingerprint also contains data relating to the database content, such as the number of subjects and clinical information. Therefore, researchers can find the databases relevant to their investigation by consulting these fingerprints.

### 3.1. Searching and Visualisation Features

Searching features over biomedical catalogues is common among medical researchers to identify databases of interest. There are currently several alternatives for searching for biomedical databases in the most common catalogues, particularly in the EMIF Catalogue. A basic search is to filter the substrings of a word. For example, a researcher who searches a database with records of patients with Alzheimer’s disease and starts by entering “alz” will see the system’s suggestions. Another form of basic search is the selection of value windows, considering a lower and upper limit.

A simple form can be used for structured searches, considering input fields operated by the logical conjunction operation. These forms contain the most relevant concepts of the fingerprints collected in each community. The main problem with this approach is that it does not consider all fingerprint concepts. The EMIF Catalogue has a different query builder to define a filter using the remaining concepts. For more complex questions, a researcher can use a form combining all possible options and disjunction operators in addition to the conjunction. The results obtained after defining such filters are presented in a result list.

The platform allows comparing a small set of databases against a reference database. Although this type of comparison is successful in some scenarios, it lacks an overview of the database network in the health domain. The EMIF Catalogue also allows selecting the question sets and databases to be exported to a spreadsheet. In this case, the researcher can use the Excel features to navigate the data, which is not user-friendly since it requires a third-party tool. The view to define this filter is a two-column list to select databases.

### 3.2. Steps towards Improved Biomedical Metadata Visualisation

The EMIF Catalogue is a platform for biomedical data discovery that adheres to FAIR (findable, accessible, interoperable, and reusable) principles [36]. The solution supports data sharing for different communities, such as the community interested in research on Alzheimer’s disease. The system uses ontologies to model the metadata to allow the annotation of several levels of information to describe the databases registered in the catalogue. The community members can annotate the concepts for the questions, and at a deeper level, they can annotate the answers to the question in the questionnaire (fingerprint). They can also have higher annotations, namely to the community itself, so the community can be related to others in the system that share the same interests.

Although we focused our initial analysis on the EMIF Catalogue, we noticed that the tabular format is the most commonly used in such platforms. Some may have charts representing specific concepts, but we identify a lack of visualisations using semantic data in health database catalogues. This fact significantly limits users’ options when selecting the databases of interest for a new research study, resulting in the reuse of databases that the researchers are familiar with instead of selecting others in the community with the potential to empower their findings.

### 3.3. Measuring User Behaviour

Quality improvement is part of the software lifecycle and can be guided by metrics and thresholds that trigger the improvement process [37]. Evaluating the information visualisations currently available in the catalogue can show how best to introduce improvements. The platform has more than 1600 registered users distributed among 11 communities. The system was initially designed to collect just a few metrics about specific views for debugging and functionality improvements. However, we considered this log helpful in providing an overview of user actions on the platform. We only used the records of the last two years for this study.

The EMIF Catalogue uses in its core the Django-Hitcount https://django-hitcount.readthedocs.io/en/latest/ (accessed on 14 September 2022) for collecting user metrics. This package counts the number of hits for a particular object in the code. For instance, the number of hits on buttons and links that open the different views that expose the information searched for by users was studied by looking at the logs. The analysis was limited to hits, since the input information defined in the filters was not recorded by this package. The existing log files do not discriminate the type of question, but rather whether or not a set of application forms was triggered, regardless of the question the researcher wants to answer. However, log files highlighted the need for new and better solutions for visualising metadata.

From the study of the logs, we concluded that users prefer to use views that allow individual dataset fingerprints to be consulted. However, the lack of comparison features in this platform and the appeals in some communities for overviews of the databases in the network motivated this work.

## 4. Methods

Visualising semantic data gives a perception of different situations and guides users in decision-making. Decisions can be based on studying database descriptions, comparing databases, and filtering and browsing data. These three search levels allow for informed decisions in conducting observational studies. In this section, we present visualisation proposals for these levels of abstraction.

### 4.1. Database-Level Visualisations

Information visualisation makes it easier to choose whether to include or exclude a database when faced with many database descriptors. Treemaps provide a visualisation of data hierarchies using nested coloured rectangular shapes. Treemaps are an alternative to visualise hierarchical structures in a compact way that allows a quick view of the relationship between the amounts of elements for each data category. When creating this type of visualisation, each category is assigned a rectangle subdivided into smaller nested rectangles representing the subcategories of data. Each rectangle size is calculated by taking the proportion of elements from each category or subcategories concerning all data. It is usual to use different colours to allow even easier reading at a glance. This type of visualisation is not suitable for ontologies that contain cycles, as it generates a recursion phenomenon that prevents the construction of the treemap.

**Figure 2:**
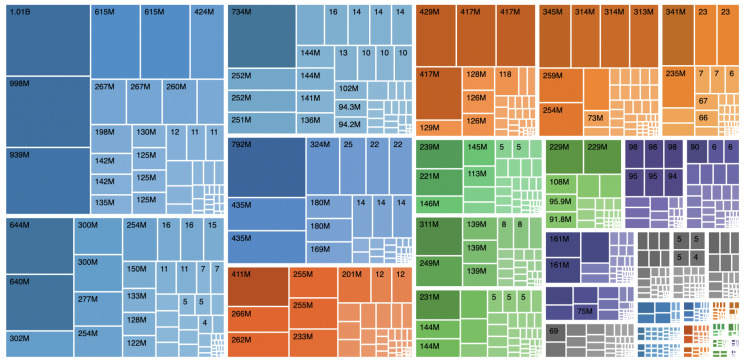
UI mockup proposal of a treemap visualisation.

The EMIF Catalogue has thousands of instances for a wide range of concepts. In addition to viewing how item percentages affect the size of rectangles, the treemap view must provide numerical information (Figure 2). When selecting one of the rectangles, more detailed information about that entity should be presented.

Semantic data find the most natural form of representation in visualisations that use graphs, since it is easy to appreciate the entities and the relationships between them [38]. However, when the number of elements increases, there is a significant loss of legibility of the presented information. The strategy to address this problem is to focus on some criterion that allows the creation of more understandable visualisations for users. Of the multiple possible criteria, our interest in visualisation at the database level aims to focus the user’s attention on that same database. Therefore, the entities and relationships related to this information must appear prominently in the foreground, introducing a differentiation that can be achieved by changing the visualised elements’ dimension and colour.

**Figure 3:**
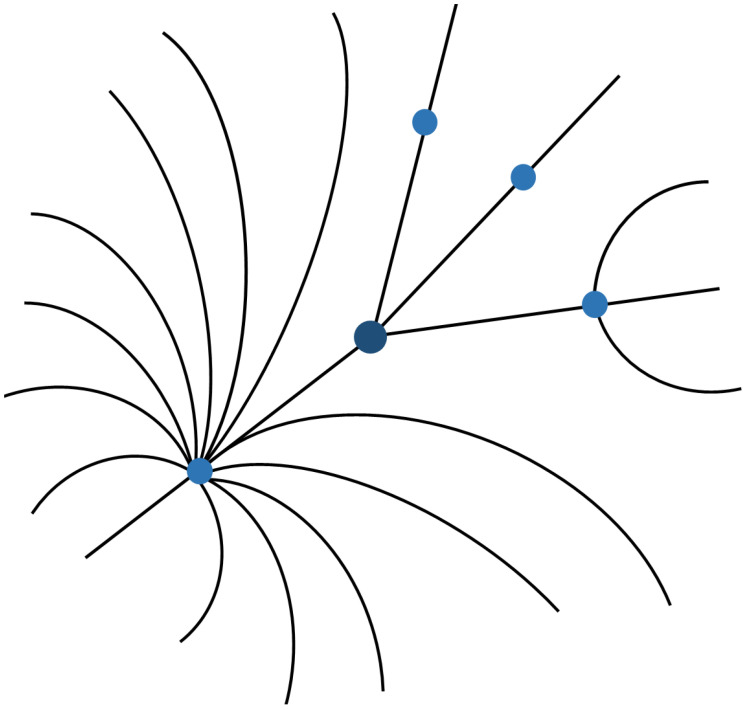
UI mockup proposal of graph visualisation.

Figure 3 shows a graph representing links between different EMIF Catalogue concepts. Each entity is represented by a point that can be clicked to obtain more detailed information. Researchers should be able to navigate the graph by selecting successive points. In this way, it is possible to perceive how the different represented instances are related.

The capture of the temporal dimension allows the study of the evolution of data classes and their instances. This leads to the need to store the history of the elements we want to monitor on the data side. The storage functionality is already implemented in the EMIF Catalogue, which keeps historical data in log files that can be accessed to operationalise the temporal visualisation of the entities of interest. From the visualisation point of view, a timeline is available for each entity or relationship clicked on by the user. There is also a snapshot of a given instant where researchers can see data at that given point in time.

**Figure 4:**
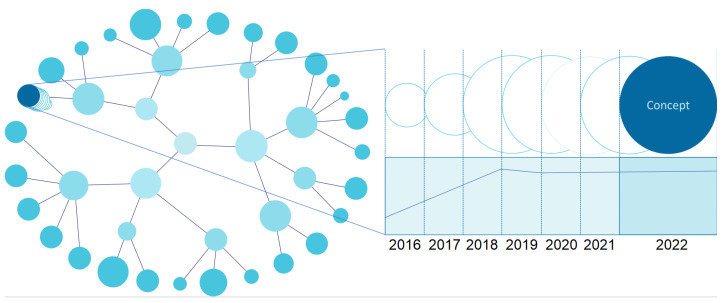
UI mockup proposal of a temporal chart visualisation (entity-level view).

For the EMIF Catalogue, there is interest in a graph-type visualisation, in which it is possible to choose any node and see its temporal evolution highlighted (Figure 4). If there is no associated historic data, only the current instance should be presented. The timeline allows navigating through different moments in time to study the state of the selected entity.

### 4.2. Network-Level Visualisations

Comparing databases allows informed choices about the data to use in medical studies. The possibility of visually comparing the contents of different data sources is an added value. It is challenging to identify the databases of interest when using tables, as is currently done in database catalogues similar to the EMIF Catalogue. The different ways of comparing semantic data related to various datasets start from graph views in an attempt to find comparisons and hierarchies.

**Figure 5:**
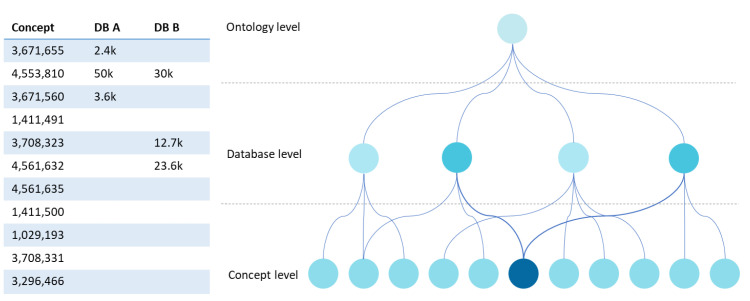
UI mockup proposal of a dendrogram visualisation.

A strategy to compare databases is to highlight hierarchical relationships extracted from the metadata. This form of structuring can be assumed when defining the ontology. In fact, “is-a” and “SubClassOf” relationships allow us to obtain dendrogram representations where we can navigate from a root node to the various branches and leaves. The right side of Figure 5 shows the dendrogram that results from processing data from the matrix on the left side of the figure. The ontology level corresponds to a central node that defines its identification and highlights the ontology’s hierarchical structure. The concepts are usually under this node, but we add an intermediate level (database level) at the network level, which allows connecting the existent concepts in each database under this ontology node. The concept level can have multiple layers. However, we simplified this idea by presenting only the leaf nodes. By simply inspecting the degree of intensity of the colour of the leaf nodes at the concept level, we can see which databases have more elements of a given concept.

Graphs are very effective for comparing concepts in different databases. Although these are good to represent entities and relationships of each database, they can also add other linked information to the visualisation that can be semantically asserted. Besides this visualisation possibility, each node or edge represented can be selected to obtain more information. As the amount of data to be presented can increase, this type of visualisation provides layers that minimise information overload and enhance the focus on relevant information. The representation of these data is similar to Figure 3, with the addition of one extra layer in the hierarchy of the ontology corresponding to each database.

**Figure 6:**
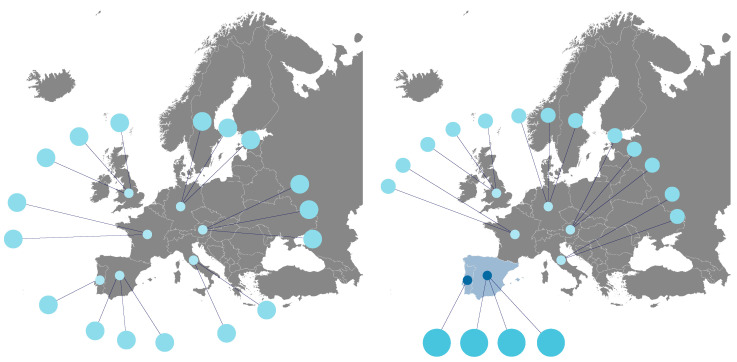
UI mockup proposal of the visualization of map charts.

Semantic visualisations can use non-traditional formats for specific domains, using images in their composition. This technique can be used when the elements of a given variable have a visual translation that simplifies the representation of the information and increases its value. An example of applying the technique is using drawings of the geographical representation of countries. In this way, we obtain illustrations of the geographic origin of the data that are much easier to grasp than the mere reading of values in tables. Figure 6 shows different databases from different European countries. On the left side of the figure, we can see the general scenario with all countries and respective databases simultaneously and with the same detail. On the right, by selecting some of the countries (e.g., Portugal and Spain), we can see highlighted the nodes that represent the databases of these countries.

Counting distinct database records allows an understanding of whether a particular choice will provide adequate data to conduct a study. Heat maps are a quick and condensed strategy to understand which databases concentrate more data on a given variable (e.g., number of patients) than previous visualisations. This type of visualisation takes two dimensions and expresses the magnitude of a given variable by gradually varying the colour. For a variable with few registers, we use a paler colour tone, and if it has many more, we use a loaded one. This graphic representation enables a quick understanding of which databases concentrate more records related to a given variable.

**Figure 7:**
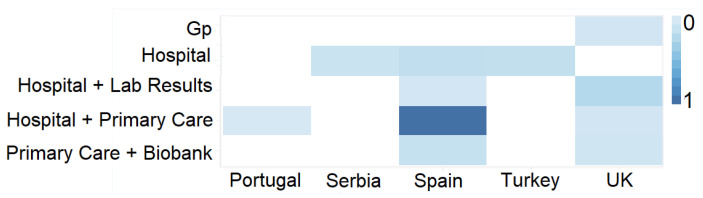
UI mockup proposal of a heat map visualisation.

Figure 7 shows an example of simplifying the cross-referenced information from databases relating to different domains and different countries of origin of the data. Darker colour tones indicate the existence of a more significant number of records. With this visualisation, researchers can quickly identify whether the data density suits their interests.

### 4.3. View Refinements

One crucial step when choosing the databases of interest to conduct a study is the stage in which the researchers need to understand the study feasibility regarding the study protocol and data available. To maximise the study’s success, this step may require several refinements since one of the main issues in medical studies is the lack of subjects with characteristics compliant with the study needs [39]. Analysing some aspects of semantic information representation more deeply allows for gaining perspective on details that might otherwise go unnoticed. The most basic way of manipulating a graphical representation of information is by zooming in on specific details of a graph.

The refinement of the information displayed can be achieved by combining it with a form for selecting values. The side-by-side view of the value refinement form and the preview pane is a powerful tool to guide users’ choices. When a node of interest is selected, researchers can make choices from a range of values and observe the impact of this choice on the visualisation being presented. In this way, they create new queries to the data and obtain the results interactively.

Visualising data with a temporal dimension should allow smooth navigation between moments in time. The chart must offer a timeline for each selectable visual element and all elements being viewed. This does not prevent the existence of static elements in time, that is, elements without historical values. In addition to the timeline, each element with history must present some overlapping representation that indicates the trend of evolution of values in the window of the closest, past, and future times, when applicable.

**Figure 8:**
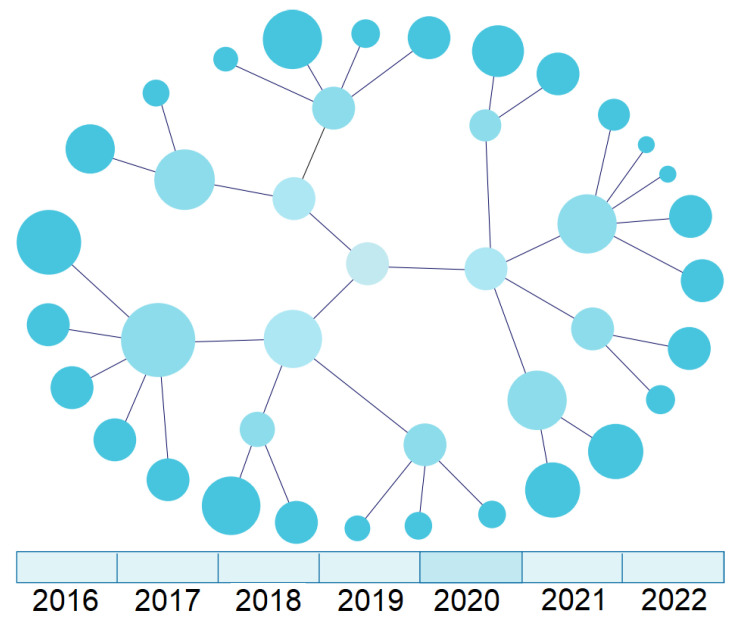
UI mockup of a temporal chart visualisation (graph-level view).

Figure 8 presents a graph-level representation with a temporal dimension. The bottom contains a timeline that allows selecting a particular year and seeing the state of the data network at that moment, as shown at the top of the figure. This feature helps a researcher navigate the different versions of database characteristics and understand the evolution of specific concepts over time, which may influence the selection of the database for the study.

We can interact with a visualisation that figuratively represents some of the semantic information nodes based on the position of these elements. The selection actions must drive a reconfiguration of the information presented, giving greater focus to the parts linked to the selected visual artefacts. It is not desirable that the components that pass into the background disappear from view, but rather that they become less prominent. One way to make selected elements stand out is to place them in a central position. Linked elements must be aligned on the bottom based on the representation to make their presence evident. The remaining secondary details can be presented in a smaller size and with more subdued colours.

Users can choose a set of countries to highlight their databases when viewing the distribution of databases by country (as shown in Figure 6). They can also select any database to see more detailed information.

## 5. Results and Discussion

To promote the quality of medical studies’ results, researchers must rely on tools that help them in each decision-making process. The availability of biomedical database catalogues is an asset for searching biomedical data. In this section, we present a proof-of-concept and discuss some typical problems in using database catalogues with visualisation features for semantic data.

### 5.1. Proof-of-Concept

Two graphical libraries based on JavaScript were used to implement a proof-of-concept and instantiate the proposed visualisations. A plugin for MONTRA was created to allow the visualisation and filtering of semantic data. For the representations of graphs and networks, Cytoscape.js https://js.cytoscape.org (accessed on 14 September 2022) was used, which enables the creation of interactive graphs for web applications. The remaining visualisations were implemented using the Highcharts https://www.highcharts.com (accessed on 14 September 2022) library. For testing, we have considered questions related to cohorts designed in the context of Alzheimer’s disease studies. Therefore, we have validated this implementation using the metadata available in the catalogue of the Alzheimer’s disease community available on the EMIF-Catalogue by testing the following questions:

A. What was the number of patients that performed a PET exam but did not perform the auditory verbal learning test?
B. What was the number of patients that performed the animal fluency test in 1 and 2 min?
C. Which patients performed attention and MRI scans?
D. Which patients that performed the Boston naming test and WAIS?
E. Which datasets have visuoconstruction and battery tests?
F. What test is recommended to detect Lewy Body Dementia?
G. Between males and females undergoing the CERAD word list exam, which had the higher scores?

We translated the questions into SPARQL queries, loading the extracted data into treemaps and heat maps. Two EMIF Catalogue users with computer science backgrounds carry out an A/B test, comparing the visualisation in the tabular format against one of the proposed visualisations freely chosen by the tester. The first question in the previous list was also filtered using geographical information and the data loaded on a map chart. The users proceeded with an in-app evaluation, rating their degree of satisfaction using a scale ranging from 1 (not at all satisfied) to 5 (extremely satisfied). Table 1 presents the average values of the collected results:

Based on the results, it is noticeable that identifying datasets of interest for conducting medical studies is more intuitive when using semantic data visualisations rather than using a tabular data solution.

### 5.2. Impact of Data Visualisations and Interactive Filtering

When researchers seek information to support a medical study, the interest is in knowing whether certain concepts are present in the available databases. In addition, they also need to know if the number of records is enough to support their work. For example, the Clinical Practice Research Datalink (CPRD) offers anonymised UK medical records, enabling the exploration of multiple dimensions such as demographics, symptoms and diagnoses [40]. The selection process is streamlined when one can see the percentages of records for each database concept at a glance. Treemaps allow the visualisation of how records are distributed by different concepts, facilitating decision-making.

To set up a cohort, researchers need to understand the relationships between the concepts to iteratively improve the selection process and move forward with confidence in the choices they make. For instance, Huang et al. [41] related epidemiological, clinical, laboratory, and radiological data to study COVID-19 treatments and outcomes. Researchers are also interested in navigating between entities and exploring the ontology’s network of connections. They also hope to interact with the visualisation to fine-tune value windows or study if a particular concept has strong connections to others. They can also use a layout that adds a value selection panel to this representation to fine-tune specific parameters. Ultimately, researchers can focus on a single node and explore in more detail the relationships it participates in for a given domain.

Temporal data often profoundly impact the quality of studies. For example, Esteban-Gil et al. [42] consider the temporal dimension in using a semantic repository of cancer patients. The availability of historical data allows for refining the data of interest and noticing trends. Visualisation facilitates the perception of hidden aspects, such as moments in time where data for a given dimension do not exist. A semantic data visualisation that allows exploring the time dimension should enable the choice of database versions that best suit the study’s design, minimising the variability derived from updates to the different data sources.

Several initiatives, such as the EMIF project or the Observational Health Data Sciences and Informatics (OHDSI) initiative [1], have highlighted the importance of comparative analyses of biomedical databases to conduct observational studies. OHDSI is an international, interdisciplinary, multi-stakeholder project to develop applications to access and analyse large-scale observational health data. The approaches described so far focus on analysing the metadata of a particular biomedical database on which researchers want to form an inclusion or exclusion opinion in a specific observational study. However, in a multicentre study, it is necessary to have a network view of the set of available databases to conduct a comparative analysis of the different options. Some authors have already contributed to this aim. For instance, the Alzheimer’s community created strategies to standardise distinct datasets and provide uniform data analysis methods [43].

Comparing two or more databases is central to performing multicentre studies, such as patient-level prediction studies. For example, Reps et al. [44] used multiple healthcare databases to reproduce two prediction models, one on type 2 diabetes and the other on dementia. A desirable way of deciding on data to train a model is, for instance, the possibility of comparatively studying the hierarchical structure of several databases. The availability of visual tools saves time and gives greater security in decision-making. In short, users have an advantage in seeing graphical representations in the form of a tree, as proposed before.

A researcher analysing database metadata may want to explore whether a particular concept is referred to in other databases. In this case, the idea is to focus on this single topic and search for it in other data sources. For instance, platforms for aggregating information on rare diseases usually collect data from distinct sources. The Diseasecard platform, which is one of these platforms, adopts graph representation and offers a navigation tree to examine networks of proteomic data and medical ontologies [45]. Considering a graph-like representation, one can select a node representing a concept for a particular database and navigate to find equivalents in another database. The visual representation of nodes and relationships is the most intuitive way to explore new data from a previously selected central concept.

The selection of data based on geographic criteria is recurrent in multicentre studies. For instance, Morales et al. [46] conducted a cohort study using electronic health records from the Information System for Research in Primary Care in Spain and the US initiatives, Columbia University Irving Medical Center Data Warehouse and the Department of Veterans Affairs Observational Medical Outcomes Partnership. The authors studied susceptibility to COVID-19 and related hospitalisation considering two cohorts, the first with patients with at least one angiotensin-converting enzyme inhibitor and angiotensin receptor blocker prescription, and the second cohort with patients using calcium channel blockers and thiazide or thiazide-like diuretics.

The proposed visualisations aim to support researchers when selecting databases of interest for a study. The EMIF Catalogue supports different database types as long as they can be represented using a set of characteristics. Our proposal only requires associating those characteristics with an ontology. Based on these conditions, the visualisation adoption by other researchers requires only a new ontology and configuring some of the visualisations to use specific attributes.

In medical studies, it is essential to have information on the types of databases per country and the number of records relating to a given concept, such as the number of patients with a given pathology. Researchers can quickly access this information using a heat map that crosses geographical data and the type of patients studied. Despite the multiple visualisation proposals discussed constituting a powerful tool to support researchers’ decision-making in their search for biomedical data, several challenges remain.

### 5.3. Open Challenges and Future Directions

Semantic data visualisation continues to raise different challenges depending on the volume of data, the complexity of ontologies, and the type of knowledge to be described. Dimensionality is critical for semantic networks with a very high number of nodes and relationships, making visualisations hard to interpret. It is necessary to explore the creation of new algorithms based on their semantic network topology to reduce the weight of dimensionality in the representation of semantic data [38]. One of the EMIF Catalogue communities, the Alzheimer’s disease community, has a structure for collecting information about datasets composed of more than 430 concepts [10]. In some views, with this number of concepts combined with a large number of registered datasets, performing a complete analysis with the traditional approaches is challenging for the researcher. However, the alternative, using a matrix view, is no better. Therefore, investing efforts in segmenting the information by adopting and implementing the visualisations described in Figure 4 and Figure 5 would increase the system’s usability.

New challenges arise when the use of multiple ontologies is required. Annotation heterogeneity leads to the need to identify different terminologies for equal entities. In health database catalogues, this is common due to the existence of many domain-specific ontologies, such as the Human Phenotype Ontology (HPO) for phenotypic abnormalities and diseases [47], the Gene Ontology (GO) for gene functions [48], and the Ontology for Biomedical Investigations (OBI) for scientific investigations [49], among others. One way forward might be to use service-oriented architectures to help efficiently discover heterogeneous datasets in other domains [50]. Promising research directions on semantic similarity in the health domain point out using ontology embeddings in supervised learning approaches [51].

Linking data over multiple semantic databases allows the creation of rich scenarios for questioning and visualising data. One can perform federated queries to obtain the desired information. However, performing federated queries remains challenging. Creating new indexing strategies and query processing schemes could be a solution [52].

Privacy in publicly released catalogue data is a related topic that sometimes goes unnoticed. There are already some algorithms to ensure data privacy for tabular data presentations [53,54,55]. However, this topic was not thoroughly studied when focusing on exposing the maximum knowledge from biomedical datasets using the proposed visualisations. Therefore, solid strategies are still needed to ensure the privacy aspects of semantic biomedical data, namely when the goal is to balance between maximum exposure, the client’s goal, and minimal disclosure of information, the provider’s concern.

## 6. Conclusions

The correct selection of databases to conduct a medical study may influence its success. Some studies could not be closed due to a lack of a substantial number of subjects. However, researchers may simplify this process using adequate strategies to represent each database’s characteristics.

In this work, a wide range of semantic data visualisations was presented as a solution to support medical researchers when creating cohorts for medical studies using catalogues of biomedical databases. The proposals intend to resolve concrete challenges in achieving this task.

The catalogue that guided our work contains metadata from biomedical databases with more than 1000 fingerprints from 11 communities registered in the system. As a result, we proposed semantic data visualisations at the database and network levels. This analysis pointed out future directions to develop new frameworks for representing semantic-based information. Although some of the proposed visualisations can be adopted using the available open-source solutions, we aimed to identify strategies that can enhance the user’s experience when selecting databases to conduct a medical study. Therefore, we have integrated the first version of such visualisations into a framework already used for exposing metadata from health databases.

## Figures and Tables

**Table 1:** Evaluation results.

Question ID	Tabular Format	Semantic Visualisation
A	3.5	5
B	2	4.5
C	4.5	5
D	3	4
E	3	4
F	2.5	3
G	3	3
A (with geo info)	1.5	4.5

## Data Availability

Not applicable.

## Abbreviations

The following abbreviations are used in this manuscript:

VQS	Visual Query System
EMIF	European Medical Information Framework
FAIR	Findable, Accessible, Interoperable, and Reusable
CPRD	Clinical Practice Research Datalink
OHDSI	Observational Health Data Sciences and Informatics
HPO	Human Phenotype Ontology
GO	Gene Ontology
OBI	Ontology for Biomedical Investigations
